# Bureau of Homœopathic Philosophy

**Published:** 1899-02

**Authors:** Wm. L. Morgan

**Affiliations:** Baltimore, Md.


### BUREAU OF HOMŒOPATHIC PHILOSOPHY.

### SOME MORBIFIC AGENCY INIMICAL TO LIFE. WHAT IS IT?

By Wm. L. Morgan, M. D., Baltimore, Md.

It is not my purpose to try to make any improvements on the works of Hahnemann, to build any new structure in therapeutics, nor to make a new materia medica. I only propose to offer a few thoughts on the relation between the cause, origin, process, and growth of disease, and its cure by the simillimum. It appears to me to be of great importance to prove that Homœopathy is a perfect system, on a scientific basis, complete in every part, and every part perfectly corresponding with all the others. My present purpose is to invite the attention of thinking people to the subject, giving a few of the reasons of my belief, with a hope that some homœopathician who has a better faculty for using words, and a larger vocabulary than I have, will take up the subject and bring it before the profession in a better manner than I am able to do. I hope my colleagues will kindly give a little attention to this subject and then judge my paper on its merits.

It is my purpose to give reasons to sustain the following aphorisms:

That disease is the result of a vital force from outside of the body—an intruder.

That the natural life and diseased life are each nourished from the surroundings outside of the body, by immaterial substances suited for each purpose.

That disease and disease-producing plants, animals, and insects are nourished and grow from the same vital principle as the simillimum.

That the natural life is nourished and sustained by an immaterial vital substance from the surroundings outside the body.

That breathing air which contains an excess of noxious gases or morbid vital substances will cause a deficiency of vital nourishment, and this deficiency will be supplied by morbid vital substances.

That an excess of morbid vitality makes food for an excess of the lower order of bacteria.

That the excess of supply over the capacity of the bacteria to absorb further displaces natural life and increases sickness. That the simillimum, by affinity, removes the morbid vitality and releases the natural life, restoring the normal condition—health.

Scientists tell us, and they generally agree on the subject, that matter is an aggregation of molecules, and that a molecule is an aggregation of atoms. That atoms composing organized matter do not touch each other, but are separated by an elastic ether. This ether, which may be regarded as a kind of vitality, varies in intensity from that in the hardest metals to that of the most volatile fluids and gases. It varies in kind and characteristics as substances vary. It controls the molecular motion, and we may claim that it controls the formation, organization, and preservation of what is called inert matter, as the natural life performs the same functions in the animal. This vitality, in its various kinds, is as necessary to the integrity of inert matter as life is to the various animals, and if withdrawn or forced away, disorganization will at once begin, just as the animal body will decompose when life is removed at death. Thus we see that this inter-atomic vitality is the life of matter and "the simillimum" of (most similar to) the natural animal life.

In the sphere of animal life we see the infinite variety and kinds of vitality from the highest type of most intelligent man downward through the various degrees of intelligence to the lowest order of parasites, including the microbe varieties, whose useful office to man is to absorb the noxious miasms that cause disease and even death to man and the higher order of animals. Hence, in looking over the vast variety of animal, bird, reptile, insect, and microbe families, we may say they are legion.

Now let us place alongside of this vast variety of organic, inorganic, fluid, and gaseous forms of matter, the equally great number of ethereal vital forces, keeping before the mind that each and every one of each kind is organized and held together by its own individual inter-atomic (vital) force, without which organic form could not exist.

The material scientist teaches the existence of atoms and molecules, and of the force that holds them together in organic form, though no man has ever seen one of them. Yet he concludes that it must be so to harmonize with other known facts.

With the same kind of reasoning we can understand that organic and inorganic substance is governed and maintained by a special vitality of its own which gives it shape or characteristics differing from all others, just as each class, species, or breed of animals differ from all others, according to its own organizing life force.

By the same reasoning the atmosphere and all gaseous matters are organized and controlled by ethereal life forces of their own kind, giving each its characteristics.

In studying the causes and conditions that lie at the foundation of sickness, we must take into consideration the soil, minerals, and topography of the section of country in which they occur. We can easily determine the locality where certain timber and kinds of vegetation will grow best, and where certain crops will yield the most. By experience and close observation it is easy to learn the healthy or sickly localities. Reptiles and the lower order of animal and insect life choose those localities in which certain vegetation grows most abundantly, for the reason that the life-nourishing ether of the region, which to man is miasmatic, producing fevers, sickness, and death, is best suited to their kind, while the reverse obtains in other places differently situated.

Take a very small seed, apparently composed of the same matter as other small seeds, but which is supplied by its parent stalk with a vital principle. While this principle is invisible to our senses, it is potent enough, when the seed is placed in favorable conditions, to start the seed to growing, and cause it to collect more material and an enlarged supply of vitality of its kind. While it is collecting, digesting, and appropriating material matter from the surroundings to build its own structure, it is also collecting from the surroundings additional vital nourishment to supply the newly acquired material with vitality of the same kind as the parent life. Where noxious plants grow and flourish to most perfect development, in the same localities man is liable to be so over-supplied with these vital plant nourishments as to render it impossible for him to get a sufficient supply of his natural vital nourishment to give force to resist the obtruding disease force. He soon absorbs too much of the latter and becomes sick. If it be of the Eupatorium, we all know of the bone aches, sick stomach, vomiting between the chill and fever in agues, found in localities where boneset grows, and how readily a case of such fever yields to Eupatorium, the simillimum.

What is true of Eupatorium will, by careful study and investigation be found applicable to every drug in the materia medica.

With regard to minerals and mineral salts, we may easily see that where they abound the decomposing processes of nature are constantly at work breaking up the organic structures and setting free the inter-atomic vitality. This floats in the air and water, or loosely combines or mixes with other substances and is absorbed, causing sickness as above mentioned.

The discovery of the X-ray gives much light on this subject. The light is formed by electricity in the ether in the Crooke's tube. In passing through the glass of the tube the light rays are refracted and the dark non-refrangible X-rays pass directly forward through various solid materials till they strike the sensitive paper, when an impression is made, but if continued too long on human tissues they produce all the blighting effect of freezing. See the application of this in nature. The sun's light comes through the ether of space. The clouds refract away the light rays, but the non-refrangible X-rays pass through the clouds and into the earth, producing many of the phenomena of the X-ray. We see, in long-continued cloudy weather, the many blighting effects on animal and vegetable life and growth, so well known to the observer, where the system is saturated with moisture to the exclusion of a pure air highly charged with vitality to nourish and give strength to the internal vital being. It has the effect of rendering the system susceptible to the encroachment of sickness of various kinds.

The late discovery of the wireless telegraph that sends its message through walls, over or through hills and rocks, and makes an intelligible impression on a sensitive receiver miles away. Those who have given much attention to the subject advance the idea that the inter-atomic ether, or whatever it may be that occupies the spaces between atoms, both in solids and fluids, has much to do as conductor or medium in transmitting this occult force to its special destination.

Sixty years ago the telegraph was not known. Thirty years ago the telephone was not known. Moving heavy cars by electricity, or lifting heavy masses of iron by magnetism was not known ten years ago.

When these discoverers mentioned their ideas they met the strongest opposition, and even ridicule from reputed scientists, until their theories were proven correct and brought into practical use. Then could I, or any one else, expect to mention the theory that the inter-atomic spaces contain the morbid vitality from the outer world (see *Organon*, Sections 11 and 18), which causes sickness, as well as the natural vital nourishment that sustains the vital powers with strength to carry on the functions of material nourishment, and that the natural life requires a constant supply of vital nourishment from without, and in the absence of that, may imbibe morbid vitality and disease without encountering the usual opposition and even unkind criticisms?

By these and many other illustrations we may understand what Hahnemann meant when he wrote Sections 11 and 18 of *The Organon*, where he referred to disease as "A morbid vitality from without, inimical to the natural life." When the system has become weakened by lack of a sufficient supply of the natural vital nourishment and is over-supplied with some morbid vitality, suited to the nourishment of the lower orders, life is obstructed, sickness and sick symptoms appear, and the morbid products in the excretions are loaded with that class of bacteria which feeds on the same morbid vital nourishment. Like all animals they congregate and multiply where the richest food is found in greatest abundance. But bacteriologists tell us that they never do harm till there is a susceptibility in the patient to their intrusion; hence they do not assemble until the food is provided by the disease vital force. Then they come; they eat, drink, and fatten, and absorb the vital poison and multiply as an evil weed absorbs its vital force. They may be collected in the excretions, triturated and potentiated and become the simillimum and curative for the same or similar sickness, like vegetable or mineral remedies, possessing the same morbid vitality.

From the above references, and by carefully reasoning from effect to cause, we may see in daily practice that sickness is caused by "A spirit-like (dynamic) process, resulting in the hurtful influences of hostile agencies from the outer world acting upon the healthy organism, and disturbing the harmonious process of life." We also may see that the bacteria have no more to do with causing sickness, either hereditary, contagious, infectious, or any other form, than the green flies that lay the eggs that make the maggots, or the buzzards and maggots that eat the carcass have to do with the killing of the animal.

